# Applying network analysis to understand the relationships between impulsivity and social media addiction and between impulsivity and problematic smartphone use

**DOI:** 10.3389/fpsyt.2022.993328

**Published:** 2022-10-18

**Authors:** Zhihua Guo, Shuyi Liang, Lei Ren, Tianqi Yang, Rui Qiu, Yang He, Xia Zhu

**Affiliations:** Department of Military Medical Psychology, Air Force Medical University, Xi’an, China

**Keywords:** social media addiction, problematic smartphone use, impulsivity, network analysis, bridge node

## Abstract

**Background:**

Prior studies have revealed the relationships between impulsivity and social media addiction (SMA) and between impulsivity and problematic smartphone use (PSU) based on total scores on standardized self-report scales. However, there has been a lack of studies exploring how the dimensions of impulsivity and components of SMA or PSU are interrelated. The present study aimed to investigate the structural relationships between the dimensions of impulsivity and components of SMA and PSU and determine the critical bridge node using network analysis.

**Methods:**

A total of 325 healthy adults aged 18–36 years participated in the study. SMA and PSU were assessed using the Bergen Social Media Addiction Scale (BSMAS) and Smartphone Application-Based Addiction Scale (SABAS), respectively. Impulsivity was measured by the Barratt Impulsiveness Scale Version 11 (BIS-11). Network analysis was used to construct an SMA-Impulsivity network and a PSU-Impulsivity network. Bridge centrality (bridge expected influence, BEI) was estimated to identify influential bridge nodes.

**Results:**

In addition to relationships within each community, network analysis revealed that the dimensions of impulsivity were closely associated with the components of SMA and PSU. Particularly, I2 “motor impulsivity” had a relatively strong connection with SMA3 “mood modification” and SMA4 “relapse” in the SMA-Impulsivity network, and with PSU2 “conflict” and PSU5 “withdrawal” in the PSU-Impulsivity network. Moreover, I2 “motor impulsivity” was identified as the most critical bridge node in both networks.

**Conclusion:**

These findings demonstrate potential pathways between different dimensions of impulsivity and the components of SMA and PSU, providing new evidence relevant to understanding the underlying mechanisms that account for how highly impulsive individuals develop SMA and PSU, and highlight the critical bridge node—motor impulsivity—that may be a promising and effective target for the prevention and treatment of SMA and PSU.

## Introduction

The addictive use of social media has grown due to the exponentially increasing use of this new technology. Social media addiction (SMA) is defined as the compulsive use of social media and a maladaptive psychological dependency on social media to the extent that addictive-like symptoms and/or reduced self-regulation occur (1–3). Some researchers also use “problematic use” to distinguish such maladaptive social media use from clinically pathological conditions (4). However, in this study, we use the term “social media addiction” because it is most commonly used in the literature (3). It has been reported that approximately 70% of Americans have used any kind of social media (5). Prior studies have revealed that SMA affects about 12% of users from different online platforms (2, 6, 7). Appropriate use of social media can facilitate communication and simplify information dissemination. However, addictive-like use (i.e., SMA) is associated with poor psychological function (8), low life satisfaction (9), low self-esteem (10), loneliness and unhealthy social relationships (1, 11), feelings of depression and anxiety (12, 13), and impulsivity (1).

With the rapid development of science and technology, smartphones have gradually become an indispensable part of daily life. According to the 47th Statistical Report on Internet Development in China issued by the China Internet Network Information Center (CNNIC), it has been reported that 99.7% of Chinese netizens (986 million) use mobile phones to access the Internet as of December 2020 and that smartphones are still the dominant device for accessing the Internet (14). Although smartphones have many advantages and can greatly facilitate people’s lives in a number of ways, such as surfing for information, communicating with others, and self-entertainment, there is growing evidence for the existence of a range of negative consequences and possible dangers associated with the problematic smartphone use (PSU) (15). PSU refers to undue attachment, a lack of self-control, and overuse when using a smartphone, which can often lead to negative consequences including physical and mental health problems (16). Both SMA and PSU are sometimes collectively referred to as Internet-related addiction (17, 18). Prior studies have shown that PSU has been linked to physical health problems, such as pain in the neck (19), head (20), and thumb (21). In addition, PSU is associated with anxiety and depression (22, 23), sleep disorders (24), loneliness (25), impulsivity (26–28), and many other psychological problems (29).

Impulsivity is variously defined as a preference for risky choices, lack of planning, the tendency to act prematurely, poor inhibition of initiated responses, and non-reflective selection of the immediately rewarding response (30–34). Accordingly, impulsivity is a multi-component rather than unitary construct (33–35). When extreme, impulsivity is an important personality trait associated with mental health problems (35). It is believed to be an important feature of destructive behavior and mental disorders such as addiction (36), paraphilias (37), personality disorders (38), and self-harm (39). Additionally, impulsivity often leads to many adverse results, including interpersonal and social problems such as aggression (40), juvenile delinquency and criminality (35), etc.

Importantly for this study, impulsivity potentially affects addictive behavior. Impulsive individuals often exhibit a lack of impulse control, which is a risk factor for different types of addictive behavior. At the same time, longitudinal studies show that there is a two-way relationship between impulsivity and addictive behavior (41). It has been shown that impulsivity is a robust predictor of substance use disorders (31, 42) as well as non-substance-related addictive disorders (43–45). As for the relationship between impulsivity and SMA, it has been reported that impulsivity is one of the most predictive factors of SMA and is tightly associated with SMA (46–52). A similar relationship exists between impulsivity and PSU. Prior studies have revealed that impulsivity plays an important role in PSU and is closely related to PSU (53–59). It is thus well established that impulsivity and Internet-related addiction (i.e., SMA and PSU) are closely interconnected; in particular, it is the case that impulsivity can develop, reinforce, and maintain the symptoms of SMA and PSU.

Prior studies have only examined the relationship between Internet-related addiction (PSU and SMA) and impulsivity using total scores on standardized instruments (46, 48, 52, 54, 57, 58). However, this common practice may obscure the specific relationships among individual symptoms and fail to reveal the interplay between the components of different scales on a fine-grained level (60, 61). In fact, impulsivity consists of three dimensions that involve different mechanisms. The three dimensions include not focusing on the task at hand (inattention), acting on the spur of the moment (motor impulsiveness), and not planning and thinking carefully (lack of planning) (30, 62). Similarly, SMA and PSU are also composed of six core factors, corresponding one-to-one to six criteria of the addiction components model, namely salience (preoccupation with the behavior), tolerance (increasing engagement in the behavior over time), mood modification (mood changes brought about by the behavior), relapse (reversion to the behavior after a period of abstinence), withdrawal (negative feelings and physical symptoms when the behavior is blocked), and conflict (interpersonal and intrapersonal relationship problems because of the behavior) (63–65). In order to facilitate our understanding of the psychopathology behind impulsivity and Internet-related addiction (SMA and PSU) and determine effective therapeutic targets, it is first necessary to investigate the interplay between the specific dimensions of impulsivity and the components of PSU and SMA.

Network analysis is a promising way to satisfy this requirement. It is a novel data-driven approach to estimating and visualizing the complex interrelations and structures of individual symptoms (60, 66), or non-symptom factors that may contribute to the development and maintenance of disorders, such as biological variables, cognitive process, behaviors, and different personality traits (67, 68). In the network theory of psychopathology, the variables dynamically interact with each other and mutually reinforce to produce a complex network, thus causing psychiatric disorders (69, 70). In the network, the various variables (i.e., symptoms, cognitive processes, and traits) are regarded as nodes; the relations (e.g., partial correlations) between observed variables are represented by node-node interactions (defined as edges) (69, 71). This approach also makes it possible to identify bridge nodes, which are nodes that strongly connect two communities (71–73). The term “community” is used to represent variables grouped together on the basis of psychological theory (73). From the perspective of a psychopathological network, bridge nodes may be considered important intervention targets. In particular, targeting bridge nodes may disrupt the connection between comorbidities and reduce the adverse effects of one disorder on others (72–75). Network analysis has been widely used to analyze co-occurring constructs, including anxiety and depression (76), posttraumatic stress disorder (PTSD) and Internet gaming disorder (77), PTSD and positive emotion dysregulation (78), and executive function and disinhibited eating (72). However, to our knowledge, there has been a lack of research using network analysis to determine the relationships between the components of Internet-related addiction (SMA and PSU) and the dimensions of impulsivity.

In order to rectify this deficiency in previous studies, we examined the interactions between Internet-related addiction and impulsivity using a network approach. First, we constructed two networks to explore the links (i.e., edges) between PSU and impulsivity and between SMA and impulsivity, respectively. We then calculated the bridge expected influence (BEI) index for each node to determine the influential bridge variables in the two symptom networks. Given that impulsivity has been robustly associated with SMA and PSU (46–59), it was hypothesized that there would be cross-community edges in addition to within-community edges. We also hypothesized that there would be variables emerging as critical bridge nodes. The purpose of this study is to identify the complex links between Internet-related addiction and impulsivity in order to deepen our understanding of the psychopathology behind them, as well as to determine effective therapeutic targets to interrupt the co-occurrence of these psychological difficulties. Given that no published studies have utilized network analysis to investigate the fine-grained relations between components of Internet-related addiction and impulsivity, our study is innovative and mostly exploratory.

## Materials and methods

### Participants and procedure

A total of 325 healthy adults aged 18–36 years were recruited to participate in the study by using convenience sampling from 27 April 2022 to 16 May 2022. We chose relatively young adults because they are more willing to embrace new technology and are predisposed to Internet-related addiction (54). The present study used an online survey based on the Wenjuanxing program.^1^ Participants gave their informed consent and were told they could withdraw from the study at any time. The anonymity of the study was emphasized to encourage honest responses in the first part of the survey. The study was reviewed and approved by the Tangdu Hospital Ethics Committee and abided by the Declaration of Helsinki.

### Measurements

#### Bergen Social Media Addiction Scale

Social media addiction was evaluated using the Bergen Social Media Addiction Scale (BSMAS) (64). The revised Chinese version of the scale was used in this study (17). The scale is comprised of six items based on six core components of the addiction components model: salience, tolerance, mood modification, relapse, withdrawal, and conflict (63). For example, item “How often during the last year used social media to forget about personal problems?” represents the “mood modification” component. Each item is rated using a 5-point Likert type scale from 1 = *very rarely* to 5 = *very often*, and the higher the score in the BSMAS, the higher the risk of developing a SMA. The internal consistency of the BSMAS in this study was fairly good (α = 0.85).

#### Smartphone Application-Based Addiction Scale

The Smartphone Application-Based Addiction Scale (SABAS) was used to assess the likelihood of being at risk of developing an addiction to PSU (79). The Chinese version of this scale was used in this study (17). The scale consists of six items that are rated on a 6-point Likert type scale ranging from 1 = *strongly disagree* to 6 = *strongly agree*. A higher score indicates a higher risk of PSU. The six items were also developed based on the perspective of the six core criteria of the addiction components model (salience, conflict, mood modification, tolerance, withdrawal, and relapse) (63, 65). For example, item “Conflicts have arisen between me and my family (or friends) because of my smartphone use” represents the “conflict” component. The Cronbach’s α coefficient for this scale was 0.83 in the current study.

#### Barratt Impulsiveness Scale Version 11

The Barratt Impulsiveness Scale Version 11 (BIS-11) is an effective self-report questionnaire employed to measure impulsivity. It consists of 30 items and is divided into three dimensions (each with 10 items): attentional impulsivity, motor impulsivity, and non-planning impulsivity (62). In the current study, we used the Chinese revised version of BIS-11 (80). Participants are asked to rate their frequency (1 = *Never*, 2 = *Rarely*, 3 = *Sometimes*, 4 = *Often*, and 5 = *Always*) for each item from motor impulsivity dimension on a 5-point Likert type scale. The dimensions of non-planning and attentional impulsivity are inverse scored items (5 = *Never*, 4 = *Rarely*, 3 = *Sometimes*, 2 = *Often*, and 1 = *Always*). The score for each dimension ranges from 0 to 100 after being converted, with higher scores indicating a higher level of impulsivity (80). The BIS-11 scale showed excellent reliability in our study (α = 0.91). Moreover, the internal consistencies of the attentional impulsivity dimension, motor impulsivity dimension, and non-planning impulsivity dimension were also fairly good (α = 0.89, 0.86, and 0.83, respectively).

### Analytical procedure

We first used SPSS (version 26.0) to perform the descriptive statistics and calculate the Cronbach’s α coefficient. We then utilized R (version 4.1.1) to perform network analysis.

The construction and visualization of the network structure were provided by the R-package *qgraph* (81). A Gaussian graphical model (GGM) was used to estimate the structure of the networks (82), including the SMA-Impulsivity network and the PSU-Impulsivity network. GGM is a type of undirected network with each edge representing the partial correlation between two nodes after statistically controlling to eliminate interference from all remaining nodes. We conducted the network structure estimation based on Spearman rho correlations (83). The GGM was estimated using the graphical least absolute shrinkage and selection operator (GLASSO) in combination with the extended Bayesian information criterion (EBIC) model selection (i.e., EBICglasso model). In detail, the GLASSO method was used to regularize the GGM (84). By shrinking all edges and pushing the trivially small partial correlation coefficients to zero, this regularization process helps to remove spurious edges and to obtain more stable and sparse networks (83, 84). To identify the optimal network model, the tuning parameter of the EBIC was set to 0.5 to balance the sensitivity and specificity of extracting true edges (83, 85). The visualization of the network layout was based on the Fruchterman-Reingold algorithm (86). Nodes with more and stronger connections with other nodes were placed closer together and more concentrated near the center of the network.

To identify bridge nodes connecting impulsivity and Internet-related addiction (SMA and PSU), we calculated the BEI using the R-package *networktools* (73). BEI is defined as the sum of the weights of all edges connecting a specific node with nodes in the other communities; BEI is especially suitable for networks with positive and negative edges, and a higher BEI value represents a higher impact on other communities (73). In our study, we pre-defined two communities in each network. We set the three impulsivity dimensions to constitute one community and the six components of SMA or PSU to form the other.

The robustness of the SMA-Impulsivity network and the PSU-Impulsivity network were evaluated using the R-package *bootnet* (86). The accuracy of the edge weights was first assessed by calculating the bootstrapped 95% confidence interval (with 1,000 bootstrap samples) for edges within the constructed networks. A narrower 95% confidence interval indicates a more reliable network (75). The stability of the node BEI was then estimated by calculating the correlation stability (CS) coefficient *via* a case-dropping bootstrap approach with 1,000 bootstrap samples. The CS coefficient was recommended to preferably be above 0.5 and never below 0.25 for enough stability (86). Finally, bootstrapped difference tests (with 1,000 bootstrap samples) were conducted for the edge weights and BEI.

## Results

### Descriptive statistics

The mean age of the participants was 21.49 ± 3.73 years (mean ± SD, range = 18–36 years). There were 147 males and 178 females in our sample. The average time the participants spent using a smartphone was 6.62 ± 3.59 h per day. Abbreviation, mean score, and standard deviation for each variable are shown in Table 1.

**TABLE 1:** Abbreviations, mean scores, and standard deviations for the study variables.

Variables	Abb	*M*	SD
**Social media addiction**			
Salience	SMA1	3.12	1.04
Tolerance	SMA2	3.01	1.10
Mood modification	SMA3	2.50	1.04
Relapse	SMA4	2.54	1.03
Withdrawal	SMA5	2.52	1.08
Conflict	SMA6	2.62	1.01
**Problematic smartphone use**			
Salience	PSU1	3.73	1.31
Conflict	PSU2	2.77	1.35
Mood modification	PSU3	3.79	1.29
Tolerance	PSU4	3.43	1.23
Withdrawal	PSU5	3.00	1.29
Relapse	PSU6	3.10	1.22
**Impulsivity**			
Non-planning impulsivity	I1	37.54	16.24
Motor impulsivity	I2	36.97	14.54
Attentional impulsivity	I3	37.46	12.72

Abb, abbreviation; *M*, mean; SD, standard deviation; SMA, social media addiction; PSU, problematic smartphone use.

### Network analysis

#### The social media addiction-impulsivity network

**FIGURE 1:**
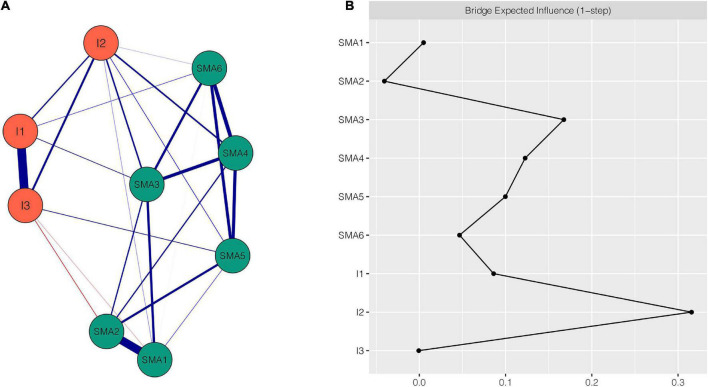
Network structure of SMA-Impulsivity variables. **(A)** EBICglasso network. Blue edges represent positive correlations, red edges represent negative correlations. A thicker edge reflects higher correlation between the nodes. **(B)** Centrality plot depicting the bridge expected influence of each node in the network (raw value). SMA1, salience; SMA2, tolerance; SMA3, mood modification; SMA4, relapse; SMA5, withdrawal; SMA6, conflict; I1, non-planning impulsivity; I2, motor impulsivity; I3, attentional impulsivity.

Figure 1A shows the SMA-Impulsivity network using the EBICglasso model. There were 9 nodes and 24 non-zero edges in the network. This network showed some characteristics as described below. First, of the five strongest edges in the network, four were between SMA components, including edges between SMA1 “salience” and SMA2 “tolerance” (weight = 0.63), between SMA4 “relapse” and SMA6 “conflict” (weight = 0.29), between SMA3 “mood modification” and SMA4 “relapse” (weight = 0.25), and between SMA4 “relapse” and SMA5 “withdrawal” (weight = 0.24); the fifth edge, between I1 “non-planning impulsivity” and I3 “attentional impulsivity,” had the strongest edge intensity (weight = 0.67). Second, some edges existed between impulsivity and SMA. I2 “motor impulsivity” showed more connecting edges with SMA. It was positively associated with five SMA components: SMA1 “salience” (weight = 0.02), SMA3 “mood modification” (weight = 0.12), SMA4 “relapse” (weight = 0.12), SMA5 “withdrawal” (weight = 0.04), and SMA6 “conflict” (weight = 0.01). Additionally, the edges between I2 “motor impulsivity” and SMA3 “mood modification” and between I2 “motor impulsivity” and SMA4 “relapse” had larger intensity than any other edges between SMA and impulsivity. Third, all edges were positive except the edges between SMA1 “salience” and I3 “attentional impulsivity” (weight = −0.02) and between SMA2 “tolerance” and I3 “attentional impulsivity” (weight = −0.04). Supplementary Table 1 shows all the edge weights within the SMA-Impulsivity network. The bootstrapped 95% confidence interval is narrow and indicates that the estimation of edge weights was relatively accurate and reliable (Supplementary Figure 1). The bootstrapped difference test for edge weights is shown in Supplementary Figure 2.

The BEI for each node within the SMA-Impulsivity network is shown in Figure 1B. Nodes I2 “motor impulsivity” (BEI = 0.31), SMA3 “mood modification” (BEI = 0.17), and SMA4 “relapse” (BEI = 0.12) all exhibited high BEI. Among them, I2 “motor impulsivity” was the most important bridge node. The CS coefficient of node BEI was 0.52, indicating that the estimations of node BEI had a good level of stability (see Supplementary Figure 3). Supplementary Figure 4 demonstrates the bootstrapped difference test for node BEI.

#### The problematic smartphone use-impulsivity network

**FIGURE 2:**
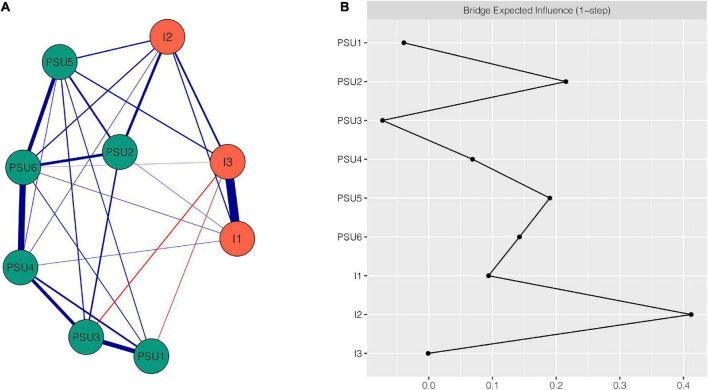
Network structure of PSU-Impulsivity variables. **(A)** EBICglasso network. Blue edges represent positive correlations, red edges represent negative correlations. A thicker edge reflects higher correlation between the nodes. **(B)** Centrality plot depicting the bridge expected influence of each node in the network (raw value). PSU1, salience; PSU2, conflict; PSU3, mood modification; PSU4, tolerance; PSU5, withdrawal; PSU6, relapse; I1, non-planning impulsivity; I2, motor impulsivity; I3, attentional impulsivity.

The PSU-Impulsivity network is shown in Figure 2A. This network featured several important characteristics. First, of the five strongest edges in the network, four were between PSU components, namely the edges between PSU4 “tolerance” and PSU6 “relapse” (weight = 0.47), between PSU1 “salience” and PSU3 “mood modification” (weight = 0.34), between PSU5 “withdrawal” and PSU6 “relapse” (weight = 0.28), and between PSU3 “mood modification” and PSU4 “tolerance” (weight = 0.24); the fifth, strongest edge was between I1 “non-planning impulsivity” and I3 “attentional impulsivity” (weight = 0.66). Second, there were some connections between impulsivity and PSU. I2 “motor impulsivity” had more connections with PSU than I1 “non-planning impulsivity” and I3 “attentional impulsivity.” I2 “motor impulsivity” was positively associated with four PSU components: PSU2 “conflict” (weight = 0.19), PSU4 “tolerance” (weight = 0.04), PSU5 “withdrawal” (weight = 0.1), and PSU6 “relapse” (weight = 0.09). In addition, the edges of I2 “motor impulsivity”-PSU2 “conflict” and I2 “motor impulsivity”-PSU5 “withdrawal” had larger intensity than any other edges between impulsivity and PSU. Third, all edges were positive except the edges between PSU3 “mood modification” and I3 “attentional impulsivity” (weight = −0.07) and between PSU1 “salience” and I3 “attentional impulsivity” (weight = −0.04). All edge weights within the PSU-Impulsivity network can be found in Supplementary Table 2. The bootstrapped 95% confidence interval indicates that the accuracy of edge weights was relatively reliable (see Supplementary Figure 5). Supplementary Figure 6 illustrates the bootstrapped difference test for edge weights.

The BEI for each node within the PSU-Impulsivity network is shown in Figure 2B. Nodes I2 “motor impulsivity” (BEI = 0.42), PSU2 “conflict” (BEI = 0.22), PSU5 “withdrawal” (BEI = 0.19), and PSU6 “relapse” (BEI = 0.15) all exhibited a high magnitude of BEI. I2 “motor impulsivity” was the most important bridge node among them. The CS coefficient of node BEI was 0.67, indicating that the node bridge centrality estimations were adequately stable (see Supplementary Figure 7). Supplementary Figure 8 shows the bootstrapped difference test for node BEI.

## Discussion

Based on network analysis of the relationships between Internet-related addiction and impulsivity, the current study not only revealed the strongest edges within each community, but also showed that the dimensions of impulsivity were correlated with the components of SMA and PSU. Moreover, this study also identified the nodes with high BEI among the SMA-Impulsivity network and PSU-Impulsivity network. To the best of our knowledge, this is the first study to utilize network analysis to investigate the relationships between impulsivity and Internet-related addiction (i.e., SMA and PSU).

Through this network analysis, we observed that the five strongest edges existed within the impulsivity community and the SMA community in the SMA-Impulsivity network, rather than connecting the two communities. Similarly, five strongest edges were found within the impulsivity community and the PSU community in the PSU-Impulsivity network. It is reasonable to expect that the strongest edges will exist between the variables within each community rather than between different communities, because the variables of each community are sub-components of the scale. These findings are similar to those of a previous study that aimed to explore the relationships between PSU (assessed by SABAS) and the Behavioral Inhibition and Activation Systems (BIS/BAS, assessed by BIS/BAS scale) using network analysis, which also showed strongest edges between components within the PSU community and the BIS/BAS community (87). These results are similar to those of other studies that investigated the relations between different communities (i.e., scales); the strong edges appeared within each symptom community rather than connecting two communities (61, 88, 89). Moreover, the current study revealed a strong connection between “non-planning impulsivity” and “attentional impulsivity.” Although previous studies have shown a positive correlation between “non-planning impulsivity” and “attentional impulsivity,” and these are both regarded as components of cognitive impulsivity (90, 91), this finding has not been explored in previous network analysis studies and needs to be further investigated.

Consistent with our hypothesis that there would be cross-community edges in addition to within-community edges, the results of the current study showed connections between the dimensions of impulsivity and the components of both SMA and PSU from a network perspective. These findings show that individuals with high impulsivity are likely to develop SMA and PSU. This is consistent with many previous studies that have shown that individuals with high trait impulsivity are prone to develop SMA and PSU (49, 50, 52–54, 92, 93). Regarding the specific dimension of impulsivity, the “motor impulsivity” dimension in impulsivity had more and stronger positive connections with components in both the SMA community and the PSU community than the other two dimensions; this explains the correlation between impulsivity and Internet-related addiction to a great extent. In detail, “motor impulsivity” showed strong and positive connections with “mood modification” and “relapse” in the SMA-Impulsivity network, while “motor impulsivity” had strong and positive connections with “conflict” and “withdrawal” in the PSU-Impulsivity network. These findings indicate that individuals with motor impulsivity are more inclined to develop some addictive components such as mood modification, relapse, conflict, and withdrawal. The connection between motor impulsivity and mood modification may result from the fact that impulse control difficulties contribute to maladaptive emotion regulation, which can lead to emotion dysregulation in response to negative life events (94, 95). As an individual’s negative emotions such as depression and anxiety build up, eventually they can lead to them engaging in the virtual world provided by Internet-related technology to regulate their mood (i.e., mood modification) (63, 87). A potential explanation for the positive associations between motor impulsivity and conflict, withdrawal, and relapse may lie in the close relationship between motor impulsivity and response inhibition. Prior studies have shown that response inhibition is closely associated with motor impulsivity (96, 97). Response inhibition refers to the ability to inhibit inappropriate or irrelevant responses, or resist temptations and resist acting impulsively so as to make flexible and goal-directed behavioral responses (98, 99). Poor inhibition and failing to resist temptations may underlie the symptoms of withdrawal and relapse, and acting impulsively may lead to conflicts with family members and friends (87, 100–102).

However, this finding does not agree with a previous study that showed that the symptom severity of SMA is mainly associated with attentional impulsivity (49). There may be two potential reasons for this discrepancy. First, different measurement instruments were used for impulsivity and SMA. The previous research used the short version of the Barratt Impulsiveness Scale (BIS-15) to evaluate impulsivity and the short Internet Addiction Test modified for social-networking sites (sIAT-SNS) to assess SMA (49). In our study, BIS-11 and BSMAS were adopted. Because the different scales were based on different models, the inter-scale relationships were also different. Second, from the methodological view, the current study utilized the network analysis method which estimates the edge strength of two nodes after considering other nodes (86), whereas the previous study used regression analyses (49). Consequently, the relationships between impulsivity and SMA in our study differ from those found in the previous study.

The bridge centrality results are consistent with our hypothesis that there would be variables emerging as critical bridge nodes. Bridge nodes can transcend the traditional psychological constructs of interest, connect theoretically independent constructs, and provide a new perspective on comorbidity (71, 73). In the current study, bridge nodes connecting dimensions of impulsivity and components of Internet-related addiction (i.e., SMA and PSU) were identified using BEI, and are crucial to understanding the development and maintenance of co-occurring psychological difficulties. They are also promising targets for prevention and intervention (70–72, 77, 88). In the SMA-Impulsivity network, the node “motor impulsivity” had the highest BEI. This indicates that “motor impulsivity” has stronger associations with components of SMA than other dimensions. Consequently, targeting the dimension “motor impulsivity” and inhibiting motor impulsivity can decrease the negative effects of motor impulsivity on SMA and thereby treat SMA. Additionally, this result implies that inhibiting motor impulsivity is a more effective way to reduce the symptoms of SMA than targeting other dimensions of impulsivity. A similar result was found in the PSU-Impulsivity network, so it is also recommended that motor impulsivity be targeted in the intervention and treatment of PSU. Coincidentally, some researchers have proposed that enhancing inhibition control could be an effective way to help decrease motor impulsivity, thereby mitigating the symptoms of SMA and PSU (2, 103). Certainly, the intervention effect of targeting motor impulsivity needs further investigation.

Although the current study provides a new perspective and fine-grained understanding of the relationships between impulsivity and SMA and between impulsivity and PSU, there are some limitations to consider. First, the study used cross-sectional data and only obtained a static network, which could not verify the dynamic changes and causality between the variables. Although the results demonstrated the important role of motor impulsivity in the prevention and treatment of SMA and PSU, we should verify the effect based on a longitudinal or experimental design in the future. Second, the results were based only on healthy young adults, and thus one should be cautious when extending the results to other age groups or clinical samples. Future studies should determine the adaptability of the results. Third, the network structure in this study was specific to the scales we used, and we utilized only BSMAS, SABAS, and BIS-11. This means that the current study did not capture all aspects of the relationships between impulsivity and SMA and between impulsivity and PSU. Consequently, it is recommended that future research analyze other aspects of the relationships between them. Fourth, using self-report data to assess both impulsivity dimensions and Internet-related addiction components is an additional limitation. Self-reports may be affected by subjective response biases, giving results that are different from the actual situation (89). This reminds us to interpret our results cautiously. Fifth, because many people may engage with social media using their smartphones, there may be an overlap between the social media use and the smartphone use. Although it did not impact the findings of this study, which investigated the fine-grained relationships between impulsivity and SMA and between impulsivity and PSU, the relationships between SMA and PSU may be worth exploring *via* network analysis in the future studies. Finally, the network structure constructed in this study was intended to examine between-subject effects on the group level, which means that the network structure may not be identical within a single individual.

## Conclusion

The present study is the first to simultaneously explore the network structure between dimensions of impulsivity and components of SMA, and between dimensions of impulsivity and components of PSU. The results elucidated some potential pathways between different dimensions of impulsivity and components of SMA and PSU. Although the strongest connections were between nodes within each scale cluster, nodes of impulsivity had strong positive associations with some components in both SMA and PSU, especially for the node “motor impulsivity.” This provides a fine-grained understanding of the psychopathological processes linking impulsivity and SMA, and linking impulsivity and PSU. Moreover, “motor impulsivity” was identified as the key bridge node which plays an important role in developing and maintaining SMA and PSU. This finding has important clinical implications, suggesting that motor impulsivity is a new and promising target to prevent or treat SMA and PSU.

## Data availability statement

The raw data supporting the conclusions of this article will be made available by the authors, without undue reservation.

## Ethics statement

The studies involving human participants were reviewed and approved by the Tangdu Hospital Ethics Committee. The patients/participants provided their written informed consent to participate in this study.

## Author contributions

ZG and XZ conceived the study. ZG, RQ, and YH performed the data collection. LR and TY performed data analysis. ZG and SL wrote the draft of the manuscript. XZ obtained funding and contributed to the manuscript revision. All authors contributed to the article and approved the submitted version.

## Footnotes

1
www.wjx.cn
